# Physico-Chemical, Sensory and Texture Properties of an Aged Mexican Manchego-Style Cheese Produced from Hair Sheep Milk

**DOI:** 10.3390/foods9111666

**Published:** 2020-11-15

**Authors:** Jesús Alberto Mezo-Solís, Víctor Manuel Moo-Huchin, Adriana Sánchez-Zarate, Manuel Gonzalez-Ronquillo, Raciel Javier Estrada-León, Rodrigo Ibáñez, Paula Toro-Mujica, Alfonso J. Chay-Canul, Einar Vargas-Bello-Pérez

**Affiliations:** 1 División Académica de Ciencias Agropecuarias, Universidad Juárez Autónoma de Tabasco, Carretera Villahermosa-Teapa, km 25, R/A. La Huasteca 2a Sección, Villahermosa CP 86280, Tabasco, Mexico; jesusmezo.solis@gmail.com (J.A.M.-S.); iq.adrianasz@gmail.com (A.S.-Z.); 2 División de Estudios de Posgrado e Investigación, TecNM-Instituto Tecnológico de Mérida, Av. Tecnológico s/n, Mérida 97000, Yucatán, Mexico; vmmoo@yahoo.com; 3 Facultad de Medicina Veterinaria y Zootecnia, Universidad Autónoma del Estado de México, Instituto Literario 100, Toluca CP 50000, Mexico; mrg@uaemex.mx; 4 TecNM-Instituto Tecnológico Superior de Calkiní, Av. Ah-Canul, Calkiní CP 24900, Campeche, Mexico; rjestrada@itescam.edu.mx; 5 Center for Dairy Research, University of Wisconsin—Madison, Madison, WI 53706, USA; ribanez@cdr.wisc.edu; 6 Instituto de Ciencias Agronómicas y Veterinarias, Universidad de O’Higgins, Campus Colchagua, San Fernando3070000, Chile; paula.toro@uoh.cl; 7 Department of Veterinary and Animal Sciences, Faculty of Health and Medical Sciences, University of Copenhagen, Grønnegårdsvej 3, DK-1870 Frederiksberg C, Denmark

**Keywords:** pelibuey ewes, sensory properties, manchego cheese, ripening, proteolysis

## Abstract

The objective of this study was to evaluate the physicochemical and texture changes of the Manchego-style cheese produced from hair sheep (Pelibuey) throughout 180 days of ripening, as well as consumer’s acceptance. Cheese pH was constant from 1 to 180 days of ripening. Moisture, water activity, fat, elasticity and hardness decreased from day 1 to day 180, while protein, trichloroacetic acid-soluble N and free amino acid increased. Cheese lightness decreased as ripening time increased, while elasticity and hardness decreased. Principal Component Analysis was useful in discriminating cheeses according to their physicochemical composition and that allowed cheeses to be classified in two groups according to their ripening time and this resulted in those with less than 60 days and those with more than 90 days of ripening. Compared with cheeses ripened at 1 and 90 days, aged cheeses at 180 days reduced scores for appearance, color, odor, taste, texture and overall acceptance. Overall, Manchego-style cheeses from hair sheep had the usual ripened-cheese physicochemical changes.

## 1. Introduction

In the past decades, research on dairy sheep has focused on the improvement of technological and coagulation properties of milk, enhancement of the nutritional value of milk to match it with dietary guidelines and production of milk as a source of components with potential benefits for human health [1]. Sheep milk is a functionally active dairy food [2] and is widely used in cheese production, mainly in Europe and some countries from Asia (China and Turkey) and the Mediterranean region where traditional dairy products such as cheeses, yoghurt, butter and ghee are manufactured [2,3].

The artisanal Mexican cheese industry has a wide variety of products, including fresh, mild and aged cheeses [4]. These cheeses are mostly based on cow’s milk but also from sheep and goat milk. In this sense, sheep milk is used in the production of ripened cheeses due to its high contents of protein, total solids and fat [5]. World-famous sheep cheese varieties are Roquefort, Feta, Teleme, Pecorino and Traditional Manchego [6,7]. These cheeses are appreciated by consumers because of their unique and delicate flavors and aromas which are in part derived from milk fatty acids [7].

In Mexico, more than 40 varieties of “artisan” cheeses have been identified, which are mainly made by small farmsteads using traditional forms of production [8]. Although Mexican artisan cheeses are mostly produced from cow’s milk, a small proportion is also made from sheep and goat milk and their mixture [4].

In recent years, an increased interest for evaluating dairy sheep production has led to studies focused on different production systems and breeds. However, there is a lack of information regarding dairy production using hair sheep (e.g., Pelibuey) under tropical conditions [9,10]. Recent studies have reported that Pelibuey sheep and their crosses yielded a milk production during their lactation from 1.1 to 1.7 kg/d under tropical conditions [10,11,12]. However, detailed data on the milk composition and its potential performance on dairy products from hair sheep is unavailable.

Unlike the traditional Spanish Manchego cheese made from sheep milk [13], the Mexican Manchego variety is a semi-hard pressed cheese made exclusively from pasteurized cow milk and ripened among 14 and 30 days, which has a mild flavor and texture [14]. We believe that milk from Pelibuey sheep has the potential to be used in the manufacture of Mexican Manchego-style cheese with good consumer’s acceptance. Therefore, the objectives of this study were to evaluate the performance of Pelibuey sheep milk production during 84 days of lactation and to evaluate changes in the composition, texture and sensory properties of Mexican Manchego-style cheese during 6 months of ripening.

## 2. Materials and Methods

### 2.1. Animals and Management

All animals were managed in compliance with the guidelines and regulations for ethical animal experimentation of the División Académica de Ciencias Agropecuarias, Universidad Juárez Autónoma de Tabasco (ID project PFI: UJAT-DACA-2015-IA-02). The experiment was carried out at the Sheep Integration Center of the Southeastern (Centro de Integración Ovina del Sureste), located in Villahermosa-Teapa, Mexico. The climate (Am) of the region is tropical humid with rains in summer, altitude is 9 m above sea level, average annual rainfall of 1958 mm, a relative humidity close to 75% and an average annual temperature of 27 °C.

Forty-two Pelibuey ewes and their lambs were confined in raised floor pens (6 × 4 m) with capacity of eight animals, where they remained until weaning. The dam diet was made using African star grass hay (*Cynodon nlemfuensis*), ground corn, soybean meal, sugarcane molasses and minerals in a proportion of 70:30 (concentrate/forage) ratio. The diet was formulated to meet the nutritional requirements of dairy ewes that had an average body weight of 45 kg and milk yield of 1.74 kg/d. The diet had 12 MJ/kg of metabolizable energy and 15% of crude protein. The amount of feed offered per pen was adjusted weekly to guarantee at least a 10% refusal. Water was available ad libitum, and health status was checked visually every day. Animals had free access to water.

### 2.2. Milk Production and Composition

The daily milk yield (DMY, kg) of ewes was recorded from 14 until 84 days post-partum. The lambs were separated daily from their dam at 19:00 h. During this period, the lambs had free access to feed (18% of crude protein, 12 MJ/kg of metabolizable energy). After 12 h of separation, ewes were manually milked after an intramuscular injection of 3 IU of oxytocin. Before milking was performed, the teats of the animals were disinfected using an iodine solution and, after about 30 s, the teats were dried with paper towels. Whole milk from all animals was pooled for cheese making.

For milk composition analysis, samples from each ewe (100 mL) were obtained weekly. Analyses for total solids, fat, protein and lactose were performed in duplicate using an automatic milk analyser (Lactoscan LS-60, Milkotronic Ltd., Nova Zagora, Bulgaria).

### 2.3. Cheese Manufacture

Mexican Manchego-style cheeses were manufactured at the Academic Division of Agricultural Sciences of the Universidad Juárez Autónoma de Tabasco, in the Dairy Products Technology Laboratory, using the protocol from Lobato-Calleros et al. [14] with some modifications. One hundred kg of milk were used for cheese making on day 35, 42 and 56. These stages of lactation were chosen since they had similar protein-to-fat ratio and lactose-to-protein. Ewe milks were weighed, filtered, pasteurized at 63 °C for 30 min and cooled to 35 °C for immediate inoculation of a mix of *Lactococcus lactis* subsp. *lactis* and *Lactococcus lactis* subsp. *cremoris* (French Bioprox M 195, Mexico) at a rate of 20 g/100 kg milk and allowed to ripen for 30 min. Milks were supplemented with a solution of calcium chloride (6% weight/volume) at 333 mL/100 kg and equilibrated for 5 min. Ten mL of commercial rennet (Cuamix, 280 International Milk Clotting Units (IMCU)/mL; Cuamex, Jalisco, México) was diluted 1:10 in potable water and added to the milk vat (100 kg), which was gently stirred for 2 min to aid dispersion. After 30 min, the coagulum was cut at similar firmness (based on the experience of the cheesemaker) using vertical and horizontal knives with 1 cm spacing between wires and healed for 5 min. The curd was then gently stirred for 10 min, followed cooking in which the curd was heated to 42 °C a rate of 1 °C/3 min and maintained at that temperature for 30 min, while the whey was completely drained from the vat during 25 min. The curd pieces were cut into small pieces by hand, dried salted with 440 g of salt, equilibrated for 20 min and placed in round stainless steel molds of 1 kg capacity. Cheeses were pressed for 24 h at 20 °C, vacuum-packaged and ripened at 10 °C and 85% relative humidity for 180 d.

### 2.4. Cheese Composition, pH and Proteolysis

Cheese samples were analyzed for moisture (oven drying method; 948.12), fat (Gerber method; 933.05), protein (% N × 6.38; Kjeldahl method; 991.20) and ash (gravimetric method; 935.42) following official AOAC methods [15], as well as salt (chloride) content by Mohr´s titration method [16]. The water activity (aw) of cheeses at 21 °C was measured using a hygrometer (Aqua Lab CX-2 Dew-Point; Decagon Devices Inc, Pullman, WA, USA). Titratable acidity (expressed as % of lactic acid) was determined with 10 g of cheese mixed with 10 mL of distilled water and titrated with 0.1 N NaOH, using 1% phenolphthalein solution in 95% ethanol as endpoint indicator (pH 8.3). The pH of cheeses at 25 °C was measured in a slurry obtained by homogenizing a mixture of 10 g of ground cheese and 10 mL of deionized water [17]. All analyses were performed in triplicate at 1, 30, 60, 90, 120, 150 and 180 d of ripening. The proteolysis of experimental cheeses during 180 d of ripening was assessed by measuring the pH 4.6-soluble N [18], 12% trichloroacetic acid-soluble N [15] and the level of total free amino acids (FAA) were determined according to the method described by Folkertsma and Fox [19]. All analyses were performed in duplicate.

### 2.5. Cheese Texture and Color

The textural properties of cheese at different time points of ripening were estimated by uniaxial compression test using an Instron Universal Testing Instrument (Model 4411 equipped with a Yoke compressor 2830-011; Instron, Canton, MA, United States) according to the method described by Vyhmeister et al. [20] with some modifications. Prior to the test, cheese cylinders (15 mm diameter and 22 mm height) were tempered at 4 °C for 24 h. Analysis was performed by compressing the samples to 70% strain at a rate of 5 mm/min. The hardness of cheeses was obtained at maximum strain. At least 10 cheese cylinders were used per sample.

The color of cheese samples was measured using a Konika-Minolta CR-300 colorimeter (Konika-Minolta Optics Inc., Osaka, Japan) according Ibañez et al. [17] using the CIELAB color system, a D65 illuminant and a visual angle of 2°. Five measurements were made on a fresh surface of cheese previously equilibrated at 20 °C for 30 min.

### 2.6. Sensory Analysis of Cheese

The sensory properties of Manchego-style cheeses were evaluated at 1, 90 and 180 d of ripening using a consumers test. Cheese cubes (2 × 2 × 2 cm^3^) at 12 °C were randomly assigned with a 3-digit code and analyzed by 75 judges using a nine-point hedonic scale (where 1 = extremely dislike; 5 = neither dislike or like; and 9 = extremely like) to evaluate attributes of appearance, color, aroma, flavor, texture and overall acceptability. Panelists were also asked to provide written additional comments from cheese samples. Judges were 24 ± 4 years old, 27 were females and 48 were males. This was approved by División Académica de Ciencias Agropecuarias, Universidad Juárez Autónoma de Tabasco (ID project PFI: UJAT-DACA-2015-IA-02).

### 2.7. Statistical Analyses

Repeated measures from 38 ewes were used for daily milk production (DMY, kg) and milk components (total solids, fat, protein, lactose, protein-to-fat and lactose-to-protein) were analyzed, adjusting a mixed linear model that included the fixed effect of litter size (LS, single or double), time (T, Production Day 14 to 84 days as a repeated measures) and the random effect of ewe (E, subject). The data analyses were performed using PROC MIXED of SAS package, version 9.3, fitting a covariance structure of compound symmetry (CS). The linear expression of the model was:

(1) $$Y_{ijk}=\mu +E_{i}+T_{j}+LS_{k}+\varepsilon_{ijk}$$

in which the abbreviations mean $\;Y_{ijklm}=$ DMY (Kg.), Fat (%), Protein (%), Lactose (%); μ = overall mean; $E_{i}$ = Random effect of i-th Ewe; $T_{j}$ = fixed effect of j-th production day; $LS_{k\;}$ = fixed effect of *k*-th litter size; $\varepsilon_{ijk}$ = residual random effects. A comparison of least square means between treatments were performed using the Tukey-Kramer test.

Data on physicochemical composition and sensory evaluation of cheeses were analyzed using a completely randomized design by analysis of variance considering the ripening times as fixed effects using the PROC GLM. Tukey’s test was performed when a significant treatment effect (*p* < 0.05) was detected. Statistical analyses were performed using the Statistical Analysis System software (SAS, 2010). In addition, selected physicochemical parameters of cheese at varying times of ripening (moisture in the non-fat substance, MNFS; fat in dry matter FDM; salt-to-moisture ratio S/M; lactic acid; pH; proteolysis (pH 4.6 SN/TN, 12% TCA SN/TN and FAA)); hardness; and CIE whiteness, L* were analyzed by principal component analysis (PCA) using a correlation matrix and a hierarchical cluster analysis (HCA) using the between groups linkage cluster method. All multivariate analyses were performed using Minitab^®^ 19 (Minitab Inc., State College, PA, USA).

## 3. Results

### 3.1. Milk Production and Composition

Daily milk production and composition of sheep milk from 14 to 84 d of lactation are detailed in Table 1. From days 14 to 21, individual sheep milk production increased from 0.43 to 0.63 kg/d, but exhibited a decrease thereafter, reaching levels of 0.26 kg/d at 84 days (*p* < 0.05). In contrast with DMY, level of TS decreased during the first 21 days to 12.4% and then increased up to 15.6% TS at 84 days of lactation (*p* < 0.001). Levels of fat, protein and protein-to-fat ratio were highly affected by time of lactation and showed a similar trend as TS (*p* < 0.05). On the other hand, levels of lactose and lactose-to-casein ratio were not affected by stage of lactation (*p* > 0.05).

### 3.2. Physicochemical Properties of Cheeses at Different Times of Ripening

The chemical composition of Manchego-style cheese made of milk from Pelibuey sheep during ripening is shown in Table 2.

**Figure 1:**
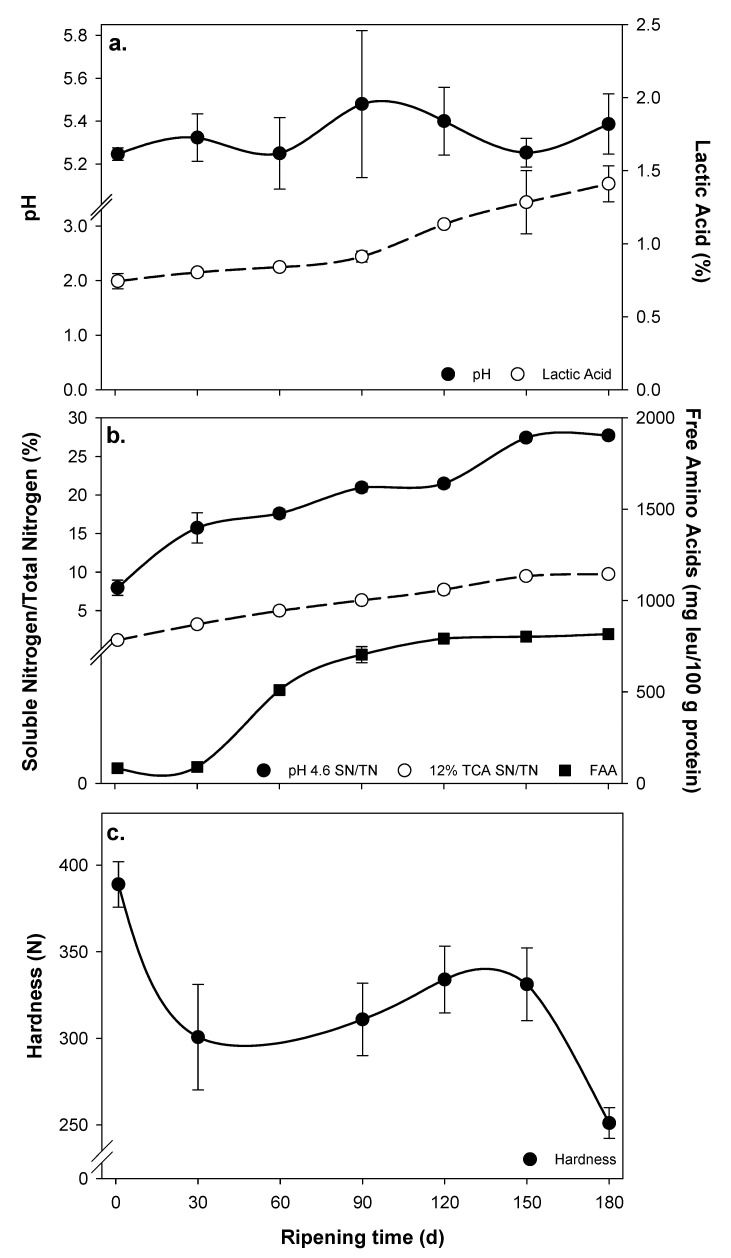
Changes in levels of pH values and lactic acid (**a**); proteolysis expressed as pH 4.6-soluble N (pH 4.6 SN/TN), 12% trichloroacetic acid-soluble N (12% TCA SN/TN) and free amino acids (FAA; (**b**)), and hardness estimated by uniaxial compression test (**c**) throughout ripening of Mexican Manchego-style cheese produced from Pelibuey ewes milk. Values represent mean and standard deviation of three replicate trials.

The moisture content during the first 60 days of ripening was constant (44%–45%) and decreased to <42% after 180 days (*p* < 0.001). A decrease in the moisture content of Manchego-style cheese during ripening led to an increase in the levels of total protein, salt, S/M and ash (*p* < 0.001) and a decrease of MNFS (*p* < 0.001). Finally, levels of fat, FDM and aw showed a decreased as ripening time progressed (*p* < 0.05). Despite that the pH values of Manchego-style cheese had a great variability and the mean values were between 5.2–5.4, they had no significant differences during ripening (*p* > 0.05); in contrast, titratable acidity (expressed as level of lactic acid) exhibited an increase (*p* < 0.05) from ~0.7% to 1.5% during 180 days (Figure 1a). Levels of primary (pH 4.6 SN/TN) and secondary proteolysis (12% TCA SN/TN) and FAA (mg leu/100 g protein) increased as ripening time of Manchego-style cheese progressed (*p* < 0.05; Figure 1b).

The color of Manchego-style cheese during 180 days of ripening is detailed in Table 3. Only whiteness, expressed by L* and WI values, exhibited a decrease after 120 days (*p* < 0.05), whereas cheese greenness (a*), yellowness (b*), chroma (C*) and hue angle (h*) remained constant as cheese aged (*p* > 0.05). However, a significant increase on color difference (∆E*) was found as cheese aged (*p* < 0.05). The instrumental hardness of cheese estimated by uniaxial compression test (Figure 1c) was highest at 1 day of ripening, then decreased at 30 days, remained constant until 150 days and finally exhibited a second decrease at 180 days (*p* < 0.05).

**Figure 2:**
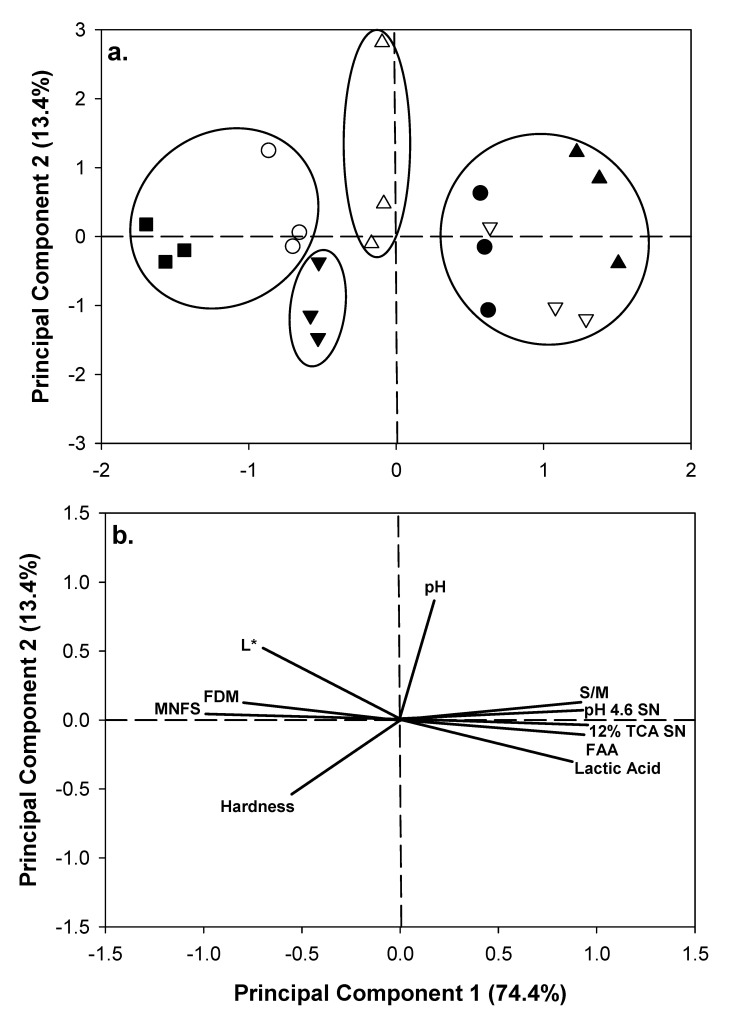
Score plot (**a**) and loading plot (**b**) obtained by principal component analysis (PCA) from selected physicochemical variables of Mexican Manchego-style cheese manufactured with hair sheep milk at 1 (■), 30 (○), 60 (▼), 90 (△), 120 (●), 150 (▽) and 180 (▲) d of ripening. Grouping of samples was based on a hierarchical cluster analysis (HCA).

A PCA performed on selected variables of cheese samples during ripening provided a simplified overview of the relationship among their physicochemical properties (Figure 2). Two components principal components (PC1 and PC2) accounted for 87.8% of total variance (74.4% and 13.4%, respectively). The score plot obtained from the two first components (Figure 2a) separated samples among ripening time for all replicate trials. Loading plots (Figure 2b) showed that the PC1 (i.e., ripening time) was negatively correlated with MNFS, FDM, hardness and WI; and positively correlated with S/M, proteolysis and lactic acid. The PC2 was positively correlated with pH values and negatively correlated with hardness. In addition, cheeses were grouped by HCA based on their ripening time: 1–30, 60, 90 and 120–180 days.

### 3.3. Sensory Properties

The sensory results of Manchego-style cheese obtained from a consumer test using a hedonic scale at different times of ripening is shown in Table 4. At 1 d of ripening, all attributes (appearance, color, aroma, flavor, texture) and overall acceptability were evaluated in the range 7–8 points (i.e., like moderately–like very much) by consumers and only a slight decrease (<1) was found for appearance at 90 days. In contrast, cheese at 180 days exhibited a significant decrease (*p* < 0.05) of all evaluated scores, although they were close to the range 6–7 (like slightly–like moderately).

## 4. Discussion

Daily milk yield in the present study was lower compared to recent reports on milk yield from hair sheep ewes. Under tropical conditions, it has been reported that Pelibuey sheep and their crosses have milk daily yields of around 1 to 1.7 kg/d [10,11,12]. However, it is important to note that daily milk yields obtained in the present study only correspond to half of the milk produced in a day. In one of the first studies that evaluated milk production and composition from Pelibuey ewes reared under tropical conditions, Castellanos and Valencia [21] proposed to use Pelibuey sheep as a dual-purpose (meat and milk production) breed. This was based on the contents of milk fat and the acceptability of sheep dairy product (especially fresh soft-cheese) compared with cow´s milk cheese in tropical areas in Mexico. The present study is the first report focused on cheese manufacturing and evaluation of the physical, chemical and sensory properties of an aged Mexican Manchego-style cheese from hair sheep ewes.

In this study, we did not perform standardization of milks prior to cheese manufacture; therefore, we selected cheeses made from milks at days 35, 42 and 56 of lactation, since they had similar protein-to-fat ratio and lactose-to-protein (Table 1) and thus led to cheeses with similar composition and acid development among replicate trials, when standard cheese manufacture protocols are applied [20]. The composition of Mexican Manchego-Style cheese made from Pelibuey sheep (Table 2) was in agreement with Mexican legislation [22] for moisture (<48%), fat (>25%), protein (>22%), salt (<3%) and pH (>5). However, ripening time led to cheeses with decreased moisture content (Table 2), which may be attributed to fermentation of lactose into lactic acid, caused by action of lactic acid bacteria, as well as increased ripening temperatures (i.e., 10 °C) [23]. Some strategies to avoid loss of moisture of cheese during ripening include controlling acid development to avoid excessive acidity, by applying curd washing/whey dilution techniques; increasing solubilization of colloidal calcium phosphate to increase capacity of proteins to retain water; and reducing ripening temperatures, since decreasing temperatures from 10 to 4 °C improves moisture retention of cheese [24]. A decrease in the moisture content of cheese during ripening led to a concomitant increase of other cheese components, such as protein, salt and ash content, which may have a great impact on texture and flavor development. In addition, a reduction of a_w_ is associated with an increase in the salt content and has a direct relation with S/M, which may modulate proteolytic and bacterial activity [25]. An increase in levels of lactic acid during the ripening of cheese could be caused by fermentation of residual lactose after manufacture [20], but also by a concomitant effect caused by a reduction of moisture content (Table 2). Solubilization of colloidal calcium phosphate caused by the presence of lactic acid leads to an increase of cheese pH during ripening [26]. Nevertheless, we observed no major changes of cheese pH during ripening, which may be influenced by the increased free amino acid content with time or may be caused by a reduction of moisture content that offset the buffering capacity by increasing levels of lactic acid (Figure 1a).

The primary proteolysis, estimated by the pH 4.6 SN/TN, is an indicator of the total peptide fraction hydrolyzed from caseins due to the action of rennet coagulant, native enzymes from milk and proteinases and peptidases from lactic acid bacteria [18]. This fraction is highly influenced by several factors, including amount of coagulant added during manufacture, acid development (chymosin activity increases at low pH values), moisture content (high MNFS leads to increased retention of chymosin), salt-to-moisture ratio (increasing levels of S/M reduce proteolysis), temperature and time of ripening (at higher ripening temperature and time, increased chymosin activity) and others [27].

Manchego-style cheese exhibited excessive proteolysis (>25% pH 4.6 SN/TN) after 120 days of ripening (Figure 1b), which could be caused by an increased retention of chymosin due to a high MNFS level and reduced S/M ratio observed on the first stages of ripening (Table 2) that enhanced chymosin activity. One of the disadvantages of excessive proteolysis in cheeses is associated with the generation of undesired flavor compounds, which are generally bitter [28]. Therefore, reducing levels of MNFS and increasing S/M are good strategies to control cheese proteolysis, as occurring in 6 months Cheddar cheeses made from cow milk [17,29]; and also reducing ripening temperatures as occurring in traditional Manchego cheese [30]. The secondary proteolysis, expressed as the 12% TCA SN/TN, correspond to the fraction of small peptides and amino acids obtained from microbial proteinases and peptidases [31]. Levels of 12% TCA SN/TN increased during ripening, but at a lower rate than primary proteolysis (Figure 1b), probably due to a reduction of MNFS and S/M levels that could have reduced activity of starter and non-starter lactic acid bacteria and their enzymes [28]. A similar explanation could also be associated with levels of FAA and its increment during ripening (Figure 1b).

A decrease in the hardness of Mexican Manchego-style cheese during ripening (Figure 1c) is caused by a softening of the para-casein matrix due to a combination of solubilization of colloidal calcium phosphate, along with an increase of proteolysis (Figure 1b) [32]. A decrease in levels of moisture and MNFS during ripening (Table 2) may have offset the softening process by showing similar hardness values among 30 and 150 days of ripening. A further softening at 180 days could be attributed to excessive proteolysis (Figure 1b).

A decrease in instrumental whiteness of cheeses, estimated by CIE L* and WI values (Table 3), is mainly associated with changes in the chemical properties of cheese, which can be caused by changes in cheese composition (a decrease in the moisture content), solubilization of colloidal calcium phosphate, increase of proteolysis as ripening progresses and an increase in pH values that modify their optical properties from opaque to translucent [20,29,33]. Dave et al. [33] found that cheeses with L* values < 85 exhibited a translucent appearance. Despite greenness, yellowness, chroma and hue angle showing no significant differences, ∆E* values increased during ripening. According to Sharma [34], ∆E* ≥ 2.3 is an indicator of a noticeable difference in color change, therefore changes in cheese color occurs at >90 days, which also agrees when they have a translucent appearance (L* < 85).

The results obtained from sensory consumer test (Table 4) agree with the findings on the physicochemical properties of Mexican Manchego-style cheese. In general, consumers gave lower scores to ripened cheeses. A decrease in color and appearance scores are in accordance with our findings on instrumental measurements of ∆E* (color difference) and whiteness (translucency), respectively (Table 3). A decrease on aroma and flavor scores as ripening time increases can be associated with the initial composition of cheeses (especially high MNFS and low S/M values; Table 2) that led to excessive proteolysis and the formation of undesired flavor and volatile compounds (peptides and products generated from catabolism of amino acids); in addition, excessive lipolysis during ripening may also generate undesired flavor compounds in cheese [30,35].

The lowest score in cheese texture at 180 days of ripening is associated with the lowest instrumental hardness (i.e., cheese extremely soft; Figure 1c) probably caused by excessive proteolysis after 150 days (Figure 1a). A decrease of overall acceptability of cheeses only at 180 days of ripening agrees with a decrease of scores in all attributes evaluated by consumers. Sensory analysis is also in agreement with the observations obtained from PCA and HCA using selected physicochemical properties of cheese (Figure 2), where cheeses were clustered in four groups (1 and 30, 60, 90 and >120 days). Cheeses from ≤90 days of ripening (i.e., mild and medium cheeses with higher sensory scores) are located on the negative side from PC1 and associate with high MNFS, low S/M, reduced proteolysis, firmer texture and whiter appearance; whereas cheeses from ≥120 days (i.e., aged cheeses with lowest sensory scores) are located on the positive side from PC1 associated with decreased MNFS, high S/M, excessive proteolysis, softer texture and translucent appearance.

Consumers sensory evaluation showed that the cheese manufacture protocol used for this study [14] is not suitable to produce an acceptable aged cheese due to its high moisture (MNFS) content and low salt (S/M) content at the beginning of ripening (1 d) that led to excessive proteolysis and thus affecting flavor and texture development. Consumer sensory evaluation also pointed at the fact that Latin American consumers prefer fresh and mild cheeses made from cow milk rather than goat and/or sheep milk [36]. Therefore, future work will be focused on modifications of cheese making protocols aiming to reduce moisture and increase salt content of Mexican Manchego-style cheese to improve physicochemical properties during aging and its acceptability from consumers.

For the first time, ripened Manchego-style cheeses manufactured from hair sheep have been characterized for physicochemical and sensory characteristics. This was an effort to provide a starting point for discussion and consideration of alternatives to the use of cow’s milk for cheese production in tropical and subtropical regions where hair sheep are available and adapt better to these environments.

## 5. Conclusions

Milk from Pelibuey sheep was successfully used in the manufacture of Mexican Manchego-style cheese with good acceptability from consumers up to 90 days of ripening. Cheeses contained a relatively high moisture and low salt content that led to development of excessive proteolysis after 120 days of ripening, which affected flavor and texture development and thus reducing sensory acceptability. Modification of cheese making protocols to adjust the cheese composition for increased aging may be a potential alternative to improve acceptability of aged Mexican Manchego-style cheese.

## Figures and Tables

**Table 1:** Pelibuey ewes milk composition and yield at different lactation stages.

Parameter	Lactation Stage (d)											SEM ^1^	*p*-Value Lactation	*p*-Value Litter Size
	14	21	28	35	42	49	56	63	70	77	84			
Yield (kg/d)	0.43	0.63	0.59	0.51	0.48	0.34	0.38	0.27	0.34	0.31	0.26	<0.01	<0.001	0.720
Total Solids (% DM)	13.32	12.40	13.23	14.57	14.36	15.45	14.54	15.01	16.10	15.70	15.56	0.60	<0.001	0.627
Fat (% DM)	2.83	2.34	2.867	4.03	4.02	4.76	4.05	4.19	5.41	4.89	5.00	0.49	<0.001	0.849
Protein (% DM)	3.11	2.83	3.09	3.48	3.43	3.73	3.47	3.50	3.92	3.83	3.78	0.18	<0.001	0.734
Lactose (% DM)	6.30	6.23	6.29	6.00	5.95	5.92	6.02	6.09	5.75	6.01	5.80	0.14	0.121	0.654
Solids Non-Fat (% DM)	10.25	9.96	10.28	10.42	10.29	10.65	10.44	10.66	10.70	10.82	10.60	0.17	0.004	0.462
Protein-to-Fat Ratio	1.38	1.58	1.43	0.98	0.95	0.88	0.93	0.98	0.77	0.88	0.77	0.11	<0.001	0.734
Lactose-to-Protein Ratio	2.25	2.44	2.26	1.87	1.88	1.76	1.85	2.40	1.51	1.55	1.50	0.32	0.228	0.660

^1^ SEM: standard error of the mean. Data is reported as mean values from 38 ewes.

**Table 2:** Physicochemical properties of Mexican Manchego-style cheese made from Pelibuey ewes milk throughout ripening.

Parameters (% Wet Basis)	Ripening Time (d)							SEM	*p*-Value
	1	30	60	90	120	150	180		
Moisture	44.69 ^a^	44.92 ^a^	44.00 ^a^	42.57 ^ab^	41.56 ^b^	41.50 ^b^	41.29 ^b^	0.464	<0.001
Fat	30.36 ^a^	28.04 ^b^	29.11 ^ab^	30.52 ^a^	29.50 ^ab^	29.21 ^ab^	28.94 ^ab^	0.430	0.021
Protein	23.92 ^d^	24.22 ^cd^	23.92 ^d^	24.61 ^c^	26.99 ^b^	27.49 ^ab^	27.79 ^a^	0.117	<0.001
Salt	1.42 ^e^	1.45 ^e^	1.52 ^d^	1.55 ^cd^	1.61 ^c^	1.69 ^b^	1.88 ^a^	0.013	<0.001
MNFS	64.17 ^a^	62.42 ^b^	62.07 ^b^	61.28 ^c^	58.96 ^d^	58.63 ^de^	58.11 ^e^	0.120	<0.001
FDM	54.86 ^a^	50.88 ^b^	51.98 ^ab^	53.15 ^ab^	50.48 ^b^	49.93 ^b^	49.29 ^b^	0.191	0.006
S/M	3.13 ^e^	3.23 ^e^	3.47 ^d^	3.57 ^d^	3.76 ^c^	4.04 ^b^	4.54 ^a^	0.031	<0.001
Ash	3.76 ^d^	4.76 ^c^	5.02 ^c^	4.82 ^bc^	5.23 ^ab^	5.22 ^ab^	5.35 ^a^	0.059	<0.001
aw	0.943 ^a^	0.944 ^a^	0.941 ^a^	0.931 ^a^	0.924 ^ab^	0.909 ^b^	0.906 ^b^	0.005	<0.001

Abbreviations are: MNFS, moisture in the non-fat substance; FDM, fat content on a dry basis weight; S/M, salt in the moisture phase of the cheese; SEM, standard error of the mean. Data are means of three replicate trials; means within a row with different superscripts show significant (*p* < 0.05) differences between ripening times.

**Table 3:** Changes in CIELAB color and whiteness index of Mexican Manchego-style cheese made from hair sheep milk at different points of ripening.

Parameters	Ripening Time (d)							SEM	*p*-Value
	1	30	60	90	120	150	180		
L*	88.65 ^a^	87.91 ^a^	86.32 ^a^	84.93 ^ab^	83.24 ^b^	81.32 ^b^	83.17 ^b^	0.545	<0.001
a*	−1.14	−1.12	−1.20	−1.26	−1.14	−1.12	−1.22	0.063	0.655
b*	17.40	17.56	17.43	16.19	17.40	16.69	16.81	0.378	0.316
C*	17.44	17.59	17.48	16.54	17.44	16.73	16.85	0.376	0.316
h* (°)	93.77	93.67	93.95	94.37	93.77	93.85	94.16	0.238	0.404
WI	36.45 ^a^	35.24 ^a^	34.02 ^ab^	35.47 ^a^	31.04 ^b^	31.25 ^b^	32.73 ^b^	1.200	0.042
∆E*	−	0.99 ^d^	2.38 ^cd^	3.85 ^bc^	5.42 ^ab^	7.42 ^a^	5.55 ^ab^	0.523	<0.001

L*, lightness or whiteness; a*, greenness or redness; b*, blueness or yellowness; h*, hue angle; C* chroma; WI, whiteness index (L* − 3b*); ∆E*, total color difference relative to color obtained at 1 d of ripening; SEM, standard error of the mean. Data are means of three replicate trials; means within a row with different superscripts show significant (*p* < 0.05) differences between ripening times.

**Table 4:** Changes in the sensory properties of Mexican Manchego-style cheese made from hair sheep milk at different points of ripening.

Attribute	Ripening Time (d)			SEM ^1^	*p*-Value
	1	90	180		
Appearance	7.8 ^a^	7.2 ^b^	6.7 ^c^	0.16	<0.001
Color	7.7 ^a^	7.0 ^ab^	6.8 ^b^	0.15	0.002
Aroma	7.2 ^a^	7.3 ^a^	6.6 ^b^	0.18	0.010
Flavor	7.3 ^a^	6.9 ^a^	5.8 ^b^	0.20	<0.001
Texture	6.7 ^ab^	7.1 ^b^	6.0 ^a^	0.21	0.001
Overall Acceptability	7.5 ^a^	7.1 ^a^	6.2 ^b^	0.17	<0.001

^1^ SEM, standard error of the mean. Data are means of three replicate trials; means within a row with different superscripts show significant (*p* < 0.05) differences between ripening times.
